# Microplastic-mediated transport of PCBs? A depuration study with *Daphnia magna*

**DOI:** 10.1371/journal.pone.0205378

**Published:** 2019-02-19

**Authors:** Zandra Gerdes, Martin Ogonowski, Inna Nybom, Caroline Ek, Margaretha Adolfsson-Erici, Andreas Barth, Elena Gorokhova

**Affiliations:** 1 Department of Environmental Science and Analytical Chemistry, Stockholm University, Stockholm, Sweden; 2 Aquabiota Water Research AB, Stockholm, Sweden; 3 Institute of Freshwater Research, Department of Aquatic Resources, Swedish University of Agricultural Sciences, Stockholm, Sweden; 4 Department of Biochemistry and Biophysics, Stockholm University, Stockholm, Sweden; VIT University, INDIA

## Abstract

The role of microplastic (MP) as a carrier of persistent organic pollutants (POPs) to aquatic organisms has been a topic of debate. However, the reverse POP transport can occur if relative contaminant concentrations are higher in the organism than in the microplastic. We evaluated the effect of microplastic on the PCB removal in planktonic animals by exposing the cladoceran *Daphnia magna* with a high body burden of polychlorinated biphenyls (PCB 18, 40, 128 and 209) to a mixture of microplastic and algae; daphnids exposed to only algae served as the control. As the endpoints, we used PCB body burden, growth, fecundity and elemental composition (%C and %N) of the daphnids. In the daphnids fed with microplastic, PCB 209 was removed more efficiently, while there was no difference for any other congeners and ΣPCBs between the microplastic-exposed and control animals. Also, higher size-specific egg production in the animals carrying PCB and receiving food mixed with microplastics was observed. However, the effects of the microplastic exposure on fecundity were of low biological significance, because the PCB body burden and the microplastic exposure concentrations were greatly exceeding environmentally relevant concentrations.

## Introduction

Microplastic (MP, particles < 5 mm) are an emerging contaminant in our environments. Concerns have been expressed that microplastic may compromise feeding of aquatic organisms and facilitate transfer of organic pollutants in food webs [1,2]. Indeed, these small plastic fragments are being ingested by a variety of organisms, e.g. fish [3], bivalves [4], polychaetes [5], and zooplankton [6], with still unknown consequences. Commonly reported effects of microplastic exposure include decreased food intake [7] and increased chemical exposure, for example, via leakage of potentially toxic additives [8] or chemicals sorbed to microplastic particles surface from ambient water [9].

Filter-feeders are vulnerable to potential impacts of microplastics. Negative effects of pristine microplastic on food intake and growth have been reported for a range of zooplankton, e.g. the cladoceran *Daphnia magna* [10] and the copepod *Calanus helgolandicus* [7], albeit at high experimental concentrations of microplastics. Moreover, for these animals at the lower trophic levels, the exposure to environmental contaminants absorbed to microplastic is of particular relevance for bioaccumulation in the food web and contaminant transfer to higher consumers. Therefore, it is important to establish whether organic and inorganic contaminants can be transferred along with microplastic to planktonic animals [11,12]. Because persistent organic pollutants (POPs), such as polychlorinated biphenyls (PCBs), are hydrophobic, they have strong partitioning towards plastic and compartments that are rich in organic carbon, e.g. biota and sediment [12]. Due to their physicochemical properties, e.g., high plastic-water partition combined with a high surface to volume ratio, microplastic particles are efficient in sorbing hydrophobic substances and may do so when the environmental conditions favour sorption [13]. Hence, microplastic may act as a vector for POPs between a consumer and its environment.

Several experimental studies that address microplastic-mediated POP transfer, from the environment to biota, were conducted using non-contaminated animals, or animals with low levels of contaminats, that were exposed to microplastics loaded with POPs [9,14–18]. Such settings are, however, rarely relevant for environmental exposure [19,20]. The ecologically plausible scenario should include consumers, microplastic and other compartments, such as natural suspensions of particulate matter, food, or sediment. Besseling et al. (2017) explored such a scenario by following PCB bioaccumulation in the lugworm *Arenicola marina*, with or without polyethylene microplastic added to the sediment [20]. In line with modelling studies [21,22], a limited microplastic effect was found–indicating that microplastic has low effect on the bioaccumulation *in situ*, as the ultimate direction of POP transport between the biota and plastic is a function of chemical fugacity gradient. In other words, if the POP concentration in the animal is higher than that in the microplastic, the contaminants will sorb to the plastic and be removed from the animal until the equilibrium is reached [23]. While this scenario is established in theory [22,23], the empirical evidence is still scarce. More experimental studies are, therefore, needed to validate the models [19] and to understand the role of microplastic in POP transport involving biota.

We explored whether exposure to microplastics (1) facilitates depuration of PCBs, similar to the sought remediation effect by adding activated carbon to contaminated sediments [24], and (2) alleviates effects of the PCB exposure on growth (somatic and reproductive) and elemental composition (carbon, %C, and nitrogen, %N) in the model filter-feeder *D*. *magna* (Straus, 1820). These effects were studied experimentally by–first–feeding *D*. *magna* neonates with PCB-contaminated food to allow for PCB accumulation in the body. Then, the animals were offered a non-contaminated food with or without microplastic addition. The changes in the somatic and reproductive growth, elemental composition and PCB concentrations of the animals were evaluated at the end of the PCB exposure (juveniles) and the end of the depuration period (egg-bearing adults). We hypothesised that (I) the addition of microplastic to the system would facilitate removal of PCB from the daphnids, and, thus, a lower PCB body burden in the microplastic-exposed daphnids will be observed, and (II) the lower PCB body burden would result in higher somatic growth, fecundity and allocation of carbon and nitrogen, in the animals exposed to microplastic compared to those fed only algae.

## Methods

### Test organisms

The freshwater cladoceran *Daphnia magna* (environmental pollution test strain *Clone 5*, the Federal Environment Agency, Berlin, Germany), were cultured in M7 media [25] at a density of 1 ind. 100 mL^-1^ and fed a mixture of green algae *Pseudokirchneriella subcapitata* and *Scenedesmus spicatus*. The algae were cultured in the MBL medium under constant light (70 μE cm^-2^ s^-1^) and temperature (24°C). Algal concentrations were determined using a 10AU Field Fluorometer (Turner Designs, Sunnyvale, California, USA) and established calibration curves.

### Polychlorinated biphenyls (PCBs)

Four PCB congeners (AccuStandard Inc., New Haven CT) covering a range of hydrophobicity within the substance group (Table 1; PCB 18, PCB 40, PCB 128 and PCB 209) were solved in toluene (AnalaR NORMAPUR VWR Chemicals) using equal proportions of each congener (by mass). This working stock was mixed with freeze-dried *Spirulina* (*Arthrospira* spp., Renée Voltaire AB, Sweden) and dried for 16 h under a stream of nitrogen to remove the toluene. The PCB-loaded *Spirulina* was suspended in 100 mL M7 and kept at +4°C before use in the feeding mixture during the accumulation phase.

**Table 1:** Log K_ow_ values and PCB concentrations in feed and *Daphnia* observed at the end of the accumulation phase and after depuration.

	Treatment	PCB 18	PCB 40	PCB 128	PCB 209
Log K_ow_^a^		5.24	5.66	6.74	8.18
*Spirulina*^b^		410 (98)	566 (75)	441 (80)	354 (69)
Accumulation phase^b*^	PCB	7.66 (0.83)	15.35 (1.53)	7.60 (1.11)	0.54 (0.20)
Depuration phase^b*^	PCB	0.54 (0.15)	1.40 (0.45)	1.37 (0.43)	0.37 (0.10)
	PCB+MP	0.47 (0.11)	1.37 (0.26)	1.50 (0.24)	0.13 (0.04)

^a^ Log K_ow_ values for PCB 18, 40 128 and 209 [30].

^b^ congener-specific PCB content, μg PCB g DW^-1^, in *Spirulina* (used as feed) and in Daphnia^*^. Values are shown as mean and SD.

### Microplastic

#### Preparation of microplastic suspension

The microplastics (fluorescent green microspheres, FMG-1.3 1–5 μm, a proprietary polymer with density of 1.3 g cm^-3^ and a melting point of 290°C) was purchased from Cospheric LLC (Goleta, USA). The polymer composition remained unidentified after FTIR Spectroscopy; see S5 Text for details on spectra and analysis. The concentration and particle size distribution of microplastics suspended in ultrapure water were measured with a laser particle counter PC-2000 (Spectrex, Redwood City, USA). The nominal size range was confirmed the nominal mean equivalent spherical diameter of 4 ± 1 μm. To prevent adherence to the surface film and homo-aggregation of microplastic, a surfactant was added at a non-toxic concentration (0.01% w/w, Tween 80, P1754 Sigma-Aldrich) [26].

### Effects of PCB and microplastic exposure on growth and elemental composition

#### Experimental outline

A two-step experimental procedure was used: (1) *accumulation phase* (day 0 to 3); the newborn daphnids were exposed to the PCB mixture via a contaminated food source, and (2) *depuration phase* (day 3 to 7); the juvenile daphnids were switched to a non-contaminated food with or without the addition of microplastic. At the termination of each phase, daphnids were sampled to measure their body size as dry weight (DW) and body length (from the centre of the eye to the base of the apical spine, BL), elemental composition (%C and %N in the body) and PCB body burden (congener-specific).

At the start of the accumulation phase, *Daphnia* (< 24 h) in groups of ten were placed in glass beakers with 500 mL M7 media (n_total_ = 71, of which 40 replicates received PCB-loaded *Spirulina*). The experiment was conducted at 22°C with a 16:8 h light: dark cycle. At the end of the accumulation phase, 12 of the control and 14 of the PCB-exposed beakers were terminated to determine the size, elemental composition and PCB body burden. The remaining beakers were divided into four treatments applied during the depuration phase: (1) *no-PCB control* with non-contaminated animals fed 100% algae (*control*, n = 11); (2) *microplastic control* with non-contaminated animals fed with the mixture of microplastic and algae (*control+MP*, n = 8); (3) PCB-loaded daphnids fed 100% algae (*PCB*, n = 13); and (4) PCB-loaded daphnids fed with the mixture of microplastic and algae *(PCB+MP*, n = 13). Upon termination of either accumulation or depuration phase, all animals were transferred in groups corresponding to replicates to new containers with 50 mL M7 media and 11 μgC mL^-1^ *P*. *subcapitata* for two hours to purge their gut contents [27] and thus minimize the impact of the chemicals originated from the bolus. The daphnids were then assigned to samples designated for either DW and elemental composition or the BL and PCB analyses.

To standardise conditions and keep particle ingestion rates constant during the experiment, the daphnids were fed *ad libitum* [28] with a food mixture of *P*. *subcapitata* and *Spirulina*, provided at 1000:1 by dry mass. To maintain the saturating food levels and a fugacity gradient that would favour depuration, the daphnids were transferred to a new media containing algae (8 μg C mL^-1^) and microplastics on days 0, 3 and 5. During the accumulation phase, the food mixture for the PCB-exposed animals contained the PCB-loaded *Spirulina*, whereas the same amount of the non-contaminated *Spirulina* was used in the control. The PCB-loaded *Spirulina* powder was added to reach the exposure concentration of 3.20 μg PCB_tot_ L^-1^ (0.74 μg L^-1^of PCB 18, 1.02 μg L^-1^ of PCB 40, 0.80 μg L^-1^ of PCB 128, and 0.64 μg L^-1^ of PCB 209). These target concentrations were determined in a pilot 48 h acute toxicity test with juvenile *D*. *magna* [25] that was conducted to establish the concentration causing < 10% mortality and measurable PCB body burden (S1 Text). The microplastic concentration during the depuration phase was 7 × 10^5^ particles mL^-1^, which equalled the particle-based algal concentration. By mass, the microplastics and algae contributed approximately 77% and 23% to the total suspended solids in the system, respectively. According to a mass balance (S2 Text), this amount of microplastic was expected to produce a measurable difference in the *Daphnia* body burden between the treatments at the end of the depuration phase.

#### Elemental analysis

The animals designated for the DW and elemental analyses were placed in pre-weighed tin capsules (4–10 individuals sample^-1^ depending on body size, n = 4–5 samples treatment^-1^), dried at 60°C for 24 h, weighed using a Sartorius M5P electronic microbalance, and stored in a desiccator. The C and N content was expressed as a mass fraction of DW (%C and %N, respectively). The analytical precision was 0.9% for %C and 0.2% for %N (S4 Text).

#### PCB analysis

All daphnids used for the PCB analysis were scanned using CanoScan 8800F flatbed scanner with ArcSoft PhotoStudio 5.5, and the images were used to determine BL (mm) and fecundity (embryos female^-1^). Using a weight-length regression established for *D*. *magna* (Eq 1) [29], the individual DW (μg) was estimated as:

$$\mathrm{DW}={1.89\times 10^{‑6}\times \mathrm{BL}}^{2.25}$$ Eq 1

For the PCB analysis, daphnids were pooled by replicate (alive individuals, ~10 sample^-1^), homogenised by bead beating (acid-washed glass beads, 212–300 μm, Sigma-Aldrich) using FastPrep (MP Biomedicals), and stored in borosilicate test tubes (-18°C) until extraction. Internal standards were used (^13^C-PCB 128 and ^13^C-PCB 209) and the samples were extracted by shaking and sonicating twice with 5 mL *n*-hexane. The extracts were pooled, and 5 ml of concentrated sulphuric acid (H_2_SO_4_) was added to remove lipids. The samples were reduced in volume (to 200 μL), and volumetric standard (PCB 52) was added before the analysis. The same PCB extraction and clean-up method was applied to PCB-loaded *Spirulina* samples. The samples (1 μL) were injected in split/splitless mode into a Thermo Scientific ISQ LR GC/MS equipped with a 30 m × 0.25 mm TG-SILMS column of 25 μm thickness. The column was held at 60°C for 2 min and then raised to 325°C at a rate of 30°C min^-1^. Compounds were analysed using positive electron ionization (EI, 70 eV) in Selected Ion Monitoring (SIM) mode. The congener mass per sample and individual DW were used to express PCB concentration; see S3 Text for details on quality assurance of the PCB analysis and the monitored ions.

#### Data analysis and statistics

A one-compartment model was used to describe the elimination behaviour of PCB from a daphnid body:

$$C_{t}=C_{o}\times {exp}^{-kt}$$ Eq 2

Where *C*_*t*_ is the concentration of a PCB congener at the time (*t*) in the depuration phase and *C*_*o*_ is the concentration at the onset of depuration (μg PCB g DW *Daphnia*^-1^). The elimination rate constant was determined by first estimating the overall elimination rate constant and then adjusting the rate constant by the growth rate constant for an individual, in order to account for growth dilution as a factor in determining the elimination rate kinetics:

$$k=k_{e}+k_{g}$$ Eq 3

Where *k* is the overall elimination rate constant (d^-1^), *k*_*e*_ and *k*_*g*_ are the final first-order elimination rate constant (d^-1^) and the growth rate (d^-1^), respectively. For each replicate, *k*_*g*_ was calculated as:

$$k_{g}=Ln\frac{{DW}_{t}}{{DW}_{0}}$$ Eq 4

where *DW*_*o*_ is the mean individual dry weight for a treatment group at the end of the accumulation phase, and *DW*_*t*_ is the mean individual dry weight of a replicate at the end of the depuration phase.

Welch’s Two Sample t-test was used to compare PCB concentrations between the *PCB* and *MP+PCB* treatments. Generalized linear models (GLMs) with log link and normal error structure were used to evaluate the effects of the PCB and microplastics exposure on the response variables (DW, BL, %C, %N, fecundity and mortality). The PCB effects on DW, %C, and %N were evaluated in juveniles at the end of the accumulation phase and in the ovigerous females at the end of the depuration phase. The data were Box-Cox transformed, and standardised residual distributions and Shapiro-Wilks test for normality were used to assess the models. Pairwise comparisons were tested using non-parametric Mann-Whitney tests. The significance level was set to α = 0.05 in all tests. S-plus 8.0 (TIBCO Software), Graphpad Prism 7.05 and R version 3.2.3 were used for the statistical tests.

## Results

### Microplastic effects on the PCB elimination rate

**Fig 1:**
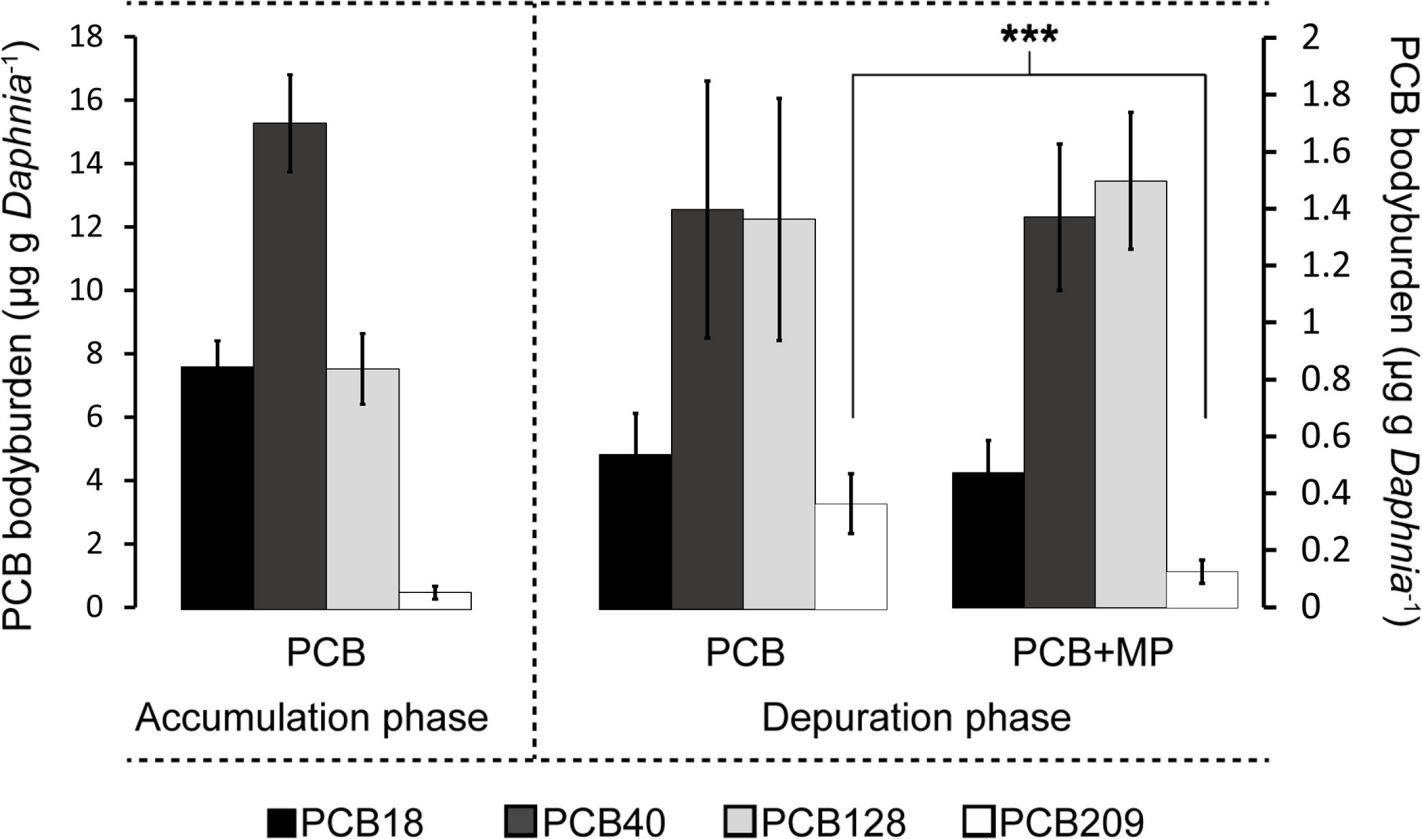
PCB Concentrations in *D*. *magna* before and after depuration. PCB concentrations (mean ± SD) in *D*. *magna* at the end of the accumulation (day 3, left y-axis), and depuration (day 7, right y-axis) phases; note the differently scaled y-axes. The concentration of PCB 209 was lower in the PCB+MP treatment compared to the PCB treatment (Welch’s Two Sample t-test, t_11_ = 5.6, p < 0.001). See also Table 1.

**Fig 2:**
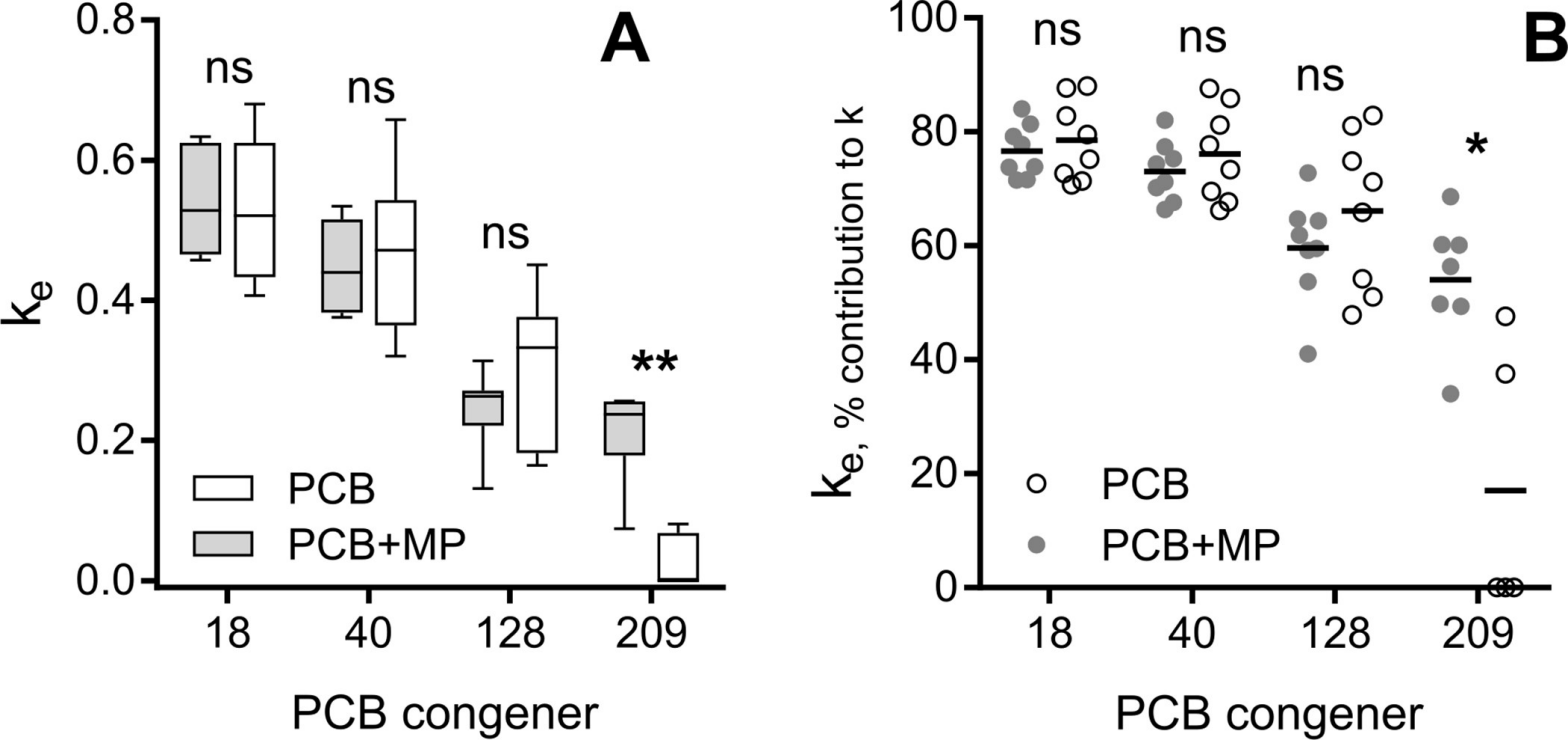
Elimination rate and the importance of growth dilution. Elimination rate constant (k_e_) for all PCB congeners (A) and contribution of k_e_ to the overall elimination rate (k) that accounts for growth dilution (B) during the depuration phase, ns: p > 0.05, *: p < 0.05, **: p < 0.01.

At the end of the accumulation phase, the total PCB body burden had reached 31.0 ± 3.6 μg g *Daphnia*^-1^ (mean ± SD, Table 1 and Fig 1), and by the end of the depuration it had declined about 10-fold; no detectable levels were observed in any of the controls. The elimination rate of the PCB congeners decreased with increasing chlorination and decreasing polarity (Fig 2A). The mean *k*_*e*_ values ranged from 0.54 d^-1^ for PCB 18 to 0.20 and 0 d^-1^ for PCB 209 in the PCB+MP and PCB treatments, respectively, with the corresponding half-lives of 1.0–1.9 and 5.7 d. A significant positive effect of microplastics on the elimination rate of PCB 209 was observed (Mann-Whitney U = 1, n_PCB_ = 7 n_MP+PCB_ = 5, p < 0.01; Fig 2A). Also, for this congener, the contribution of *k*_*e*_ to the overall elimination rate constant *k* was significantly higher in the PCB+MP group (Mann-Whitney, U = 2, n_PCB_ = 7 n_MP+PCB_ = 5, p < 0.05, Fig 2B). For the other congeners, *k*_*e*_ contributed ~60–80% to their *k* values (Fig 2B), with higher values for congeners with lower logK_OW_ and no differences between the treatments.

### Single and combined PCB and microplastic effects on the life history traits

#### Mortality

In all treatments, mortality was low, not exceeding 7% during the accumulation phase and 8% during the depuration phase across the treatments. Exposure to microplastic did not affect mortality (GLM, Z_3,6_ = 0.10, p > 0.99 and Z_3,6_ = -0.10, p > 0.99 for the MP × PCB interaction and MP, respectively).

#### PCB and microplastic effects

**Fig 3:**
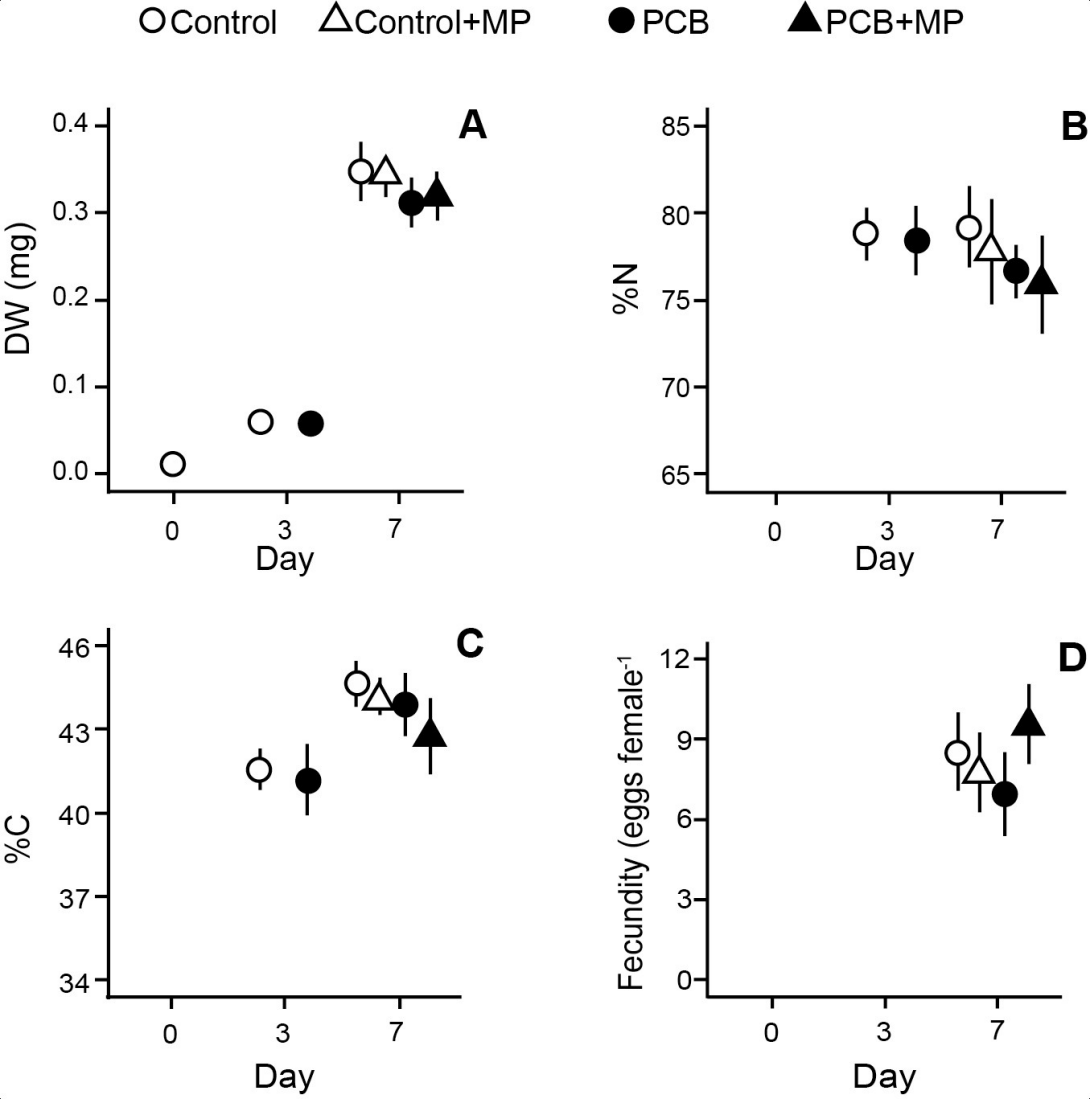
Treatment effects on the life-histories and body composition of *D*. *magna*. The life-history parameters and body composition on days 0, 3 (post accumulation phase) and 7 (post depuration phase): (A) individual body mass (DW), (B) carbon (%C) and (C) nitrogen content (%N), and (D) fecundity (eggs female^-1^). No eggs had been produced at the termination of the accumulation phase. Data are shown as mean ± SD. Some error bars are invisible due to the low SD.

**Fig 4:**
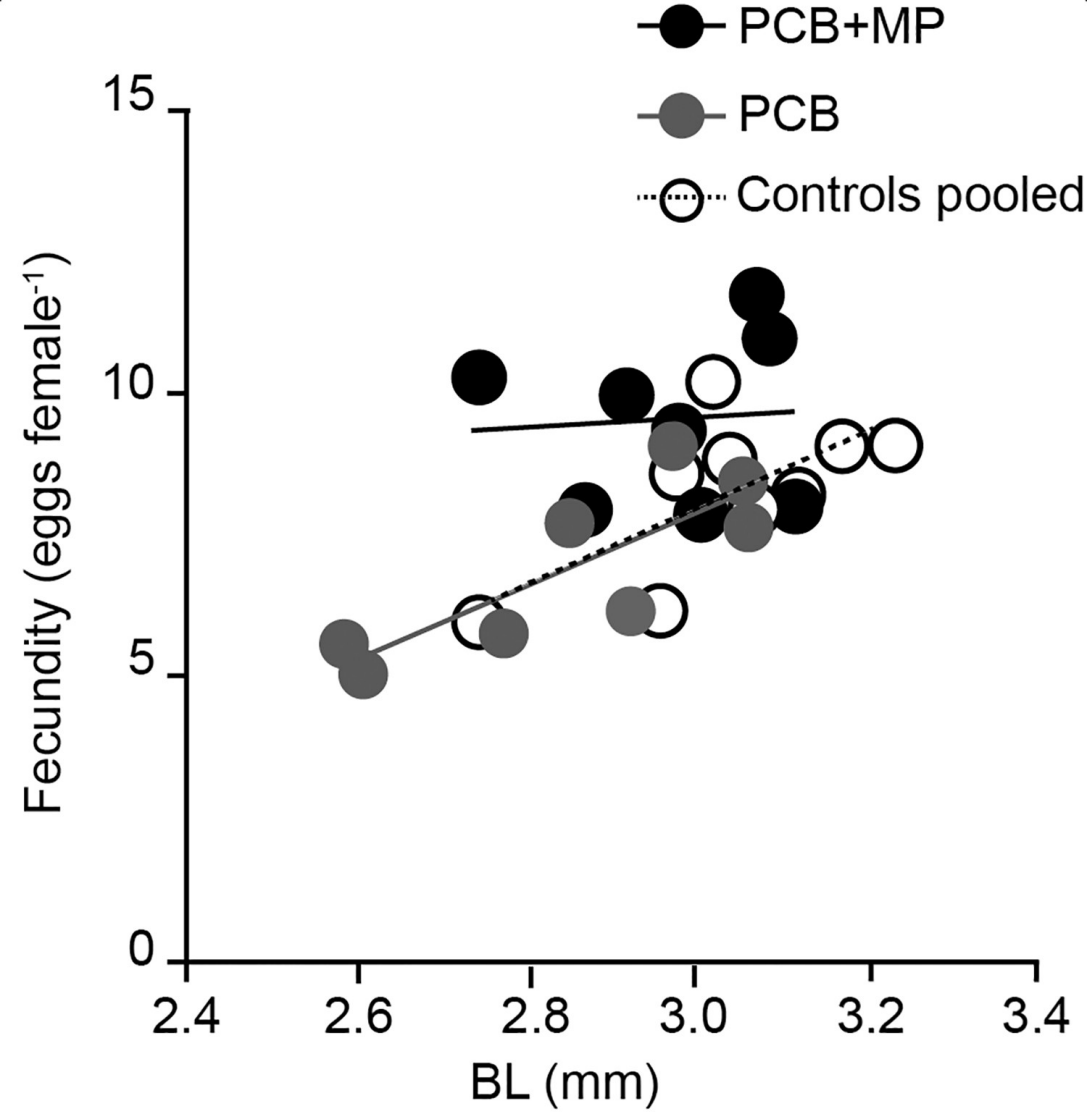
Treatment-specific relationships between fecundity and body length in *D*. *magna*. Whereas the overall relationship was significant, the group-specific slope was significantly different from zero only in the PCB group (R^2^ = 0.7, p < 0.02) and in the pooled controls (R^2^ = 0.5, p < 0.05).

At the end of the accumulation phase, no significant effects of the PCB exposure on the body size, %C or %N in the juveniles were observed (Table 2, Fig 3A–3C). At the end of the depuration phase, the PCB-exposed animals were significantly smaller (by 10% and 4% for DW and BL, respectively), whereas no microplastic effect on either DW or BL was observed (Table 2, Fig 3A). For all PCB-exposed daphnids, there was a significant negative relationship between the BL and PCB 209 concentration in the adults (GLM, Wald Stat._1, 11_ = 6.0, p = 0.01). Moreover, the %N values were significantly lower in the PCB-exposed daphnids compared to the control, with no additional effect exerted by microplastic (Table 2, Fig 3B). The %C values were also negatively affected by PCB exposure; furthermore, the %C values were significantly lower in the microplastic-exposed daphnids (Table 2, Fig 3C). The fecundity was positively related to the BL, with a significant positive effect of microplastic on the intercept of this relationship in the PCB-exposed animals (Fig 4, GLM, Wald Stat. _4, 9_ = 8.2, p < 0.01). This interaction implies higher size-specific egg production in the animals carrying PCB and receiving food mixed with microplastics. Fecundity in the PCB+MP treatment was also elevated in comparison to the control treatment receiving microplastic (control+MP, Fig 3D, GLM, t_1, 14_ = 3.3, p < 0.01).

**Table 2:** Summary of generalized linear models results.

Phase	Dependent variable	Explanatory variable	Estimate	SE	Wald Stat.	p-value
Accumulation	DW	PCB	-0.077	0.216	0.126	0.722
	%C	PCB	-0.005	0.007	0.400	0.508
	%N	PCB	-0.009	0.030	0.090	0.762
Depuration	DW	MP	-0.005	0.023	0.053	0.818
		PCB	-0.060	0.023	6.721	**0.009**
		PCB × MP	-0.012	0.023	0.252	0.616
	BL	MP	-0.014	0.010	1.870	0.171
		PCB	-0.022	0.010	4.620	**0.032**
		PCB × MP	-0.007	0.010	0.420	0.518
	Fecundity	MP	-0.147	0.114	1.682	0.194
		PCB	0.014	0.114	0.014	0.904
		PCB × MP	-0.280	0.114	6.063	**0.014**
	%C	MP	0.000	0.000	4.700	**0.029**
		PCB	0.000	0.000	6.700	**0.010**
		PCB × MP	0.000	0.000	0.500	0.459
	%N	MP	0.034	0.031	1.170	0.279
		PCB	-0.072	0.031	5.220	**0.022**
		PCB × MP	-0.009	0.031	0.080	0.078

GLMs testing treatment effects (PCB and MP against the control animals) and their interactions on the body size (DW, BL), fecundity, and elemental composition (%C and %N) in *D*. *magna* at the end of the accumulation and depuration phases. Significant effects are in bold.

## Discussion

The role of plastic debris as a carrier of POPs to aquatic biota has been much debated, but most of the evidence suggests that the bioaccumulation is mainly governed by the ingestion of contaminated prey [31]. Here, we tested whether microplastic can increase the transport of PCBs from microscopic crustaceans with short gut residence time using a scenario that included both food and microplastic in the exposure system. According to the theoretical mass-balance of our depuration system (S2 Text), we anticipated a higher PCB 209 elimination in the daphnids receiving microplastic compared to those fed only algae. This was indeed the case, whereas there was no effect on the other congeners and the ΣPCBs despite the high amount of the plastic in the experimental system. Thus, our first hypothesis that microplastics can increase PCB elimination in daphnids was confirmed, albeit only for the high-molecular-weight congeners.

The positive relationship between hydrophobicity of the PCBs and the contribution of growth to its elimination (Fig 2B) was expected, as more hydrophobic substances have longer half-lives [32]. The observed half-lives (~1–5 days) are reasonable based on literature, particularly when considering the relative importance of body surface area and volume for equilibration time. For example, longer half-lives were reported for PCBs in larger crustaceans, e.g. 11 d for PCB 153 in the shrimp *Palaemonetes varians* [33] and 30 d for a PCB-mixture (PCB 40–81) in the amphipod *Diporeia spp*. [34]. In the copepod *Acartia tonsa* with a body size slightly smaller than that in *D*. *magna*, half-lives of 0.5–1 d for Aroclor 1254 (mainly penta and hexa-chlorinated biphenyls [35]) were reported [36].

The slower elimination of PCB 209 compared to the other congeners was most pronounced in the microplastic-free treatment, resulting in a half-life of 4.8 d, whereas addition of microplastic decreased it to 2.0 d (Fig 2A). This difference indicates that our microplastic was effective sorbent in the experimental setting. However, the commonly reported microplastic concentrations *in situ* are much lower than those used in our study [37], and environmental zooplankton samples contain 20-fold to four orders of magnitude less PCBs than our post-depuration daphnids [38–40]. Therefore, any environmentally plausible microplastic effects on the elimination rates in zooplankton would be low. However, in oligotrophic plastic accumulation zones, a high proportion of plastic to organic material has been reported [41]. Therefore, one can speculate that in such environments fugacity gradients may favour plastic-organism transfer for certain chemicals but this remains to be tested.

Our experiment was designed as a proof-of-principle to test whether depuration via microplastics can occur. Using fish as a model organism, a similar depuration study has been conducted by Rummel and colleagues (2016). However, due to high variability in their fish’s depuration, their results were inconclusive regarding whether there were any changes in PCB elimination rate by plastic in the fish diet [42]. To the best of our knowledge, an indication of plastic acting as a sink has only been seen in accumulation studies, where POP uptake is often reduced in the presence of microplastic [e.g., 16]. While the relative importance of microplastic for bioaccumulation of POPs, compared other matrices in the aquatic environment, is considered small, exploring contaminant transfer is relevant for understandning microplastic hazard potential. Future studies should focus on linking the physiochemical properties of the microplastic, identifying those essential for predicting polymer sorptive capacity [43,44], and, account for the microplastic effects on test organsisms biochemical composition due to, e.g., changes in food quality. There is, thereby, still a need in experimental data to quantify POP dynamics in systems containing microplastic for improving our understanding of their role in bioaccumulation of various contaminants, by different organisms, and at different pollution levels [19,44].

Our second hypothesis that lowered PCB body burden in the microplastic-exposed daphnids would result in higher fitness was also partially supported. The addition of microplastic to the food did not affect animal growth, whereas the reproductive output increased. However, the positive effect of microplastic on the daphnid reproduction was only significant in combination with PCB exposure and coincided with the higher elimination of PCB 209 in the daphnids exposed to the microplastic. Overall, the PCB effects observed in the experiment were consistent with the relatively low toxicity of non-dioxin-like PCBs in daphnids [45].

Interestingly, the daphnids fed microplastic-algae mixture had 1% lower carbon content, while no effect on the %N content was observed, which may indicate subtle effects on the energy budget and higher energy allocation to maintenance as well as changes in body stiochiometry. However, without analysis of the biochemical composition, particularly lipid and protein content, this difference is difficult to attribute to a specific physiological change. An increased allocation to reproductive growth is often linked to higher %C content as eggs contain more carbon per dry mass than somatic tissues [46]. However, no individual-level responses, such as fecundity or growth, coincided with the elevated %C content (Fig 3C and 3D). One can speculate, that the higher fecundity observed in the PCB-challenged daphnids exposed to microplastics led to changes in their metabolism, e.g., more extensive use of lipids [47]. As proteins are relatively low in carbon compared to the lipids, these changes can imply alterations in the elemental composition, such as the decrease in %C levels of somatic tissues and eggs. Moreover, a higher number of embryos in the brood under PCB-induced stress would increase embryonic respiration losses, which may lead to lower %C of the bulk biomass [48].

An alternative explanation to the higher size-specific fecundity in PCB-contaminated daphnids exposed to microplastic is that this occurred as an adaptation to a compromised food quality, because nutrient content per particle was lower in the microplastic algae mixtures. Mixtures with non-palatable particles are likely to be perceived by the daphnids as a poor quality food, which may lead to a preferential investment in progeny rather than to somatic tissues [49,50]. In this case, a trade-off can manifest as an increased production of smaller offspring, a commonly observed cladoceran response to deteriorated feeding environment [48]. Such responses have been observed in zooplankton that reduced filtering rates at high abundances of inert particles [7,51]. In combination with PCB contamination, these poor feeding conditions may have caused a counterintuitive response manifested as the simultaneous decrease in %C content and the increase in fecundity. Unfortunately, it was not feasible to measure the offspring size, which would have helped to understand the combined effects of microplastics and contaminant exposure on the elemental composition and the life histories in this experiment.

The interplay between growth, elemental composition and PCB body burden in the microplastic-exposed daphnids is intriguing and provides insights into the experimental design and informative endpoints to measure. Body burden of hydrophobic chemicals is a function of lipid content in the organism, including zooplankton [52]. Effects on the lipid metabolism would, therefore, affect contaminant accumulation, but also growth performance, which may differ ontogenetically as well as among species. Various effects of nanoparticles and microplastics on lipid metabolism have been reported in fish [53] and invertebrates [54,55], with both increased and decreased lipid accumulation. Microplastics have also been found to affect lipid metabolism in zebrafish [56]. Thus, the observed changes in %C content in the daphnids may also indicate alterations in lipid metabolism. Such changes suggest that the lipidome profile and biomarkers of lipid synthesis pathways can be of interest in microplastic effect studies, together with the involved physiological (respiration, reproduction, assimilation efficiency and offspring quality) and stoichiometric (C:N and C:P ratios) responses.

## Conclusions

Considering that reported environmental concentrations of microplastics are orders of magnitude lower than those used in our study [56] and that microplastics contribute very little to the ambient concentrations of suspended solids, their *in situ* effects on PCB body burden, growth, and reproduction in zooplankton are most likely negligible. However, the exposure of PCB-contaminated daphnids to microplastic facilitated elimination of the high-molecular-weight PCB by 4-fold compared to the animals receiving only algal food, which provides proof-of-principle that microplastics can act as a sink for hydrophobic organic contaminants. Although subtle, the decreased carbon content may indicate important metabolic and stoichimetric changes in the animals exposed to microplastic. Given the ecological importance of daphnids (and primary consumers at large) in nutrient recycling, these changes may represent an important mechanism of ecosystem-level responses. Future studies need to focus on the improved mechanistic understanding of the microplastic effects across the biological organization levels.

## Supporting information

S1 Text Establishing PCB exposure concentrations. (DOCX)

S2 Text Mass-balance calculations for PCBs in the test system. (DOCX)

S3 Text Quality assurance for PCB analysis. (DOCX)

S4 Text Quality assurance for elemental composition. (DOCX)

S5 Text Polymer characterization by Fourier Transform Infrared (FTIR) Spectroscopy. (DOCX)
