# Supplementary material for: Microplastic-mediated transport of PCBs? A depuration study with Daphnia magna
Source: PLoS One. 2019 Feb 19;14(2):e0205378. doi: 10.1371/journal.pone.0205378 (PMC6380591; doi:10.1371/journal.pone.0205378)
Supplement: S1 Text — (DOCX) [file pone.0205378.s001.docx]

# S1 Text Establishing PCB exposure concentrations

Two tests were conducted to (1) find a target PCB exposure concentration and (2) confirm that *D. magna* body burden is above the quantification limit. The 48 h Acute Immobilisation Test [1] for *Daphnia sp.* was combined with a feeding rate test, 24 h post-exposure. The test concentration range (Table A) was based on the study by Lynn and colleagues [2] who exposed *Daphnia pulicaria* to PCB 54.

## Method

On the first day of the immobilization test, the daphnids (10 individuals, 500 mL M7 test medium, 3 replicates per concentration; Table A) were fed a mixture of PCB-loaded *Spirulina* and *Pseudokirchneriella subcapitata* (8 μg C mL^-1^). To determine the body burden, five animals per replicate were used for the PCB analysis, and the remaining five were used in the post-exposure feeding rate test to assess sub-lethal effects. The assessment of possible alterations in feeding rate was needed to ensure that filtering and intake of particles were not compromised in the PCB-exposed animals. In the feeding rate test, the daphnids were held in beakers with 50 mL M7 and 1.5 μg C mL^-1^ *P. subcapitata* during 24 h. Beakers were kept in darkness at 22° C to limit algal growth. Feeding rate was calculated as the reduction in the algal concentration, which was measured using a fluorometer (Turner designs, 10-AU) at 0 h and 24 h.

## Results

None of the tested concentrations affected the feeding rate. The concentration 3.2 μg total PCB L^-1^ resulted in 10% mortality, while the body burden was sufficient for PCB analysis (Table A). The lack of effects on the feeding rate in the exposed animals compared to the control supported the choice of approximately 3.2 μg total PCBs L^-1^ as a reasonable exposure level.

**Table A. PCB exposure, pilot experiment results.**

| **Exposure** | **Daphnids exposure response and accumulated body burden at 48h** | | | | | | | |
| --- | --- | --- | --- | --- | --- | --- | --- | --- |
| PCB_tot_  (μg g^-1^) | DW  (mg) | PCB 18  (μg g^-1^) | PCB 40  (μg g^-1^) | PCB 128  (μg g^-1^) | PCB 209  (μg g^-1^) | ΣPCB  (μg g^-1^) | Mortality | Feeding rate  μg C h^-1^ |
| 0 | 0.55 (0.11) | NF | NF | NF | NF | NF | 0% (0) | 0.71 (0.01) |
| 0.4 | 0.43 (0.07) | NF | 0.94 (0.49) | 0.90 (0.19) | 0.07 (0.09) | 1.9 (0.65) | 3% (5) | 0.77 (0.02) |
| 0.8 | 0.59 (0.05) | 0.61 (0.12) | 2.42 (0.10) | 2.34 (0.04) | NF | 5.37 (0.01) | 10% (9) | 0.74 (0.03) |
| 1.6 | 0.62 (0.10) | 0.75 (0.30) | 3.24 (0.82) | 2.16 (0.35) | NF | 6.15 (1.46) | 10% (0) | 0.79 (0.01) |
| 3.2 | 0.75 (0.07) | 0.87 (0.21) | 4.79 (0.81) | 3.93 (0.60) | NF | 9.58 (1.53) | 10% (0) | 0.74 (0.01) |
| 32 | 0.78 (0.06) | 7.69 (3.08) | 34.88 (8.69) | 29.04 (5.72) | 1.37 (0.33) | 72.99 (17.77) | 20% (8) | 0.72 (0.01) |

Congener-specific *Daphnia* PCB-concentration (µg g^-1^) and DW (mg) of the juveniles after 48 h exposure, as well as the results of the acute test (% mortality) and the post exposure feeding rate test (feeding rate μg C h^-1^). Values are shown as mean and SD. The congeners’ relative contribution to PCB_tot_ exposure was PCB 18: 0.23, PCB 40: 0.32, PCB 128: 0.25, and PCB 209: 0.20.

# References

1. OECD. Test No. 202: *Daphnia sp*. Acute Immobilisation Test [Internet]. Paris: Organisation for Economic Co-operation and Development; 2004.

2. Lynn SG, Price DJ, Birge WJ, Kilham SS. Effect of nutrient availability on the uptake of PCB congener 2,2′,6,6′-tetrachlorobiphenyl by a diatom (*Stephanodiscus minutulus*) and transfer to a zooplankton (*Daphnia pulicaria*). Aquat Toxicol. 2007;83: 24–32.
