# Supplementary material for: Microplastic-mediated transport of PCBs? A depuration study with Daphnia magna
Source: PLoS One. 2019 Feb 19;14(2):e0205378. doi: 10.1371/journal.pone.0205378 (PMC6380591; doi:10.1371/journal.pone.0205378)
Supplement: S2 Text — (DOCX) [file pone.0205378.s002.docx]

# S2 Text. Mass-balance calculations for PCBs in the test system

In our closed system, the PCB water concentration is dependent on the relative concentration of PCB in food, MP and *Daphnia*. Consequently, the addition of non-contaminated MP should decrease PCB concentration in *Daphnia*. To anticipate whether our experimental setup can produce differences between the treatments and whether MP can act as a sink [1], we conducted a mass balance calculation. For the purposes of the calculation a thermodynamic equilibrium was assumed. At the end of experiment the system was expected to approach equilibrium, as in similar systems an equilibrium has been reported to occur within 36h for a range of PCB congeners [2,3]. Thus, the calculated PCB concentrations in equilibrium were expected to be close to the measured concentrations at the end of the experiment.

PCB 209 was used in the mass balance because of its high hydrophobicity that makes it more likely to be affected by MP. The distribution of PCB 209 during the depuration phase was calculated for an experimental system without microplastic (water, algae and daphnids) and with microplastic (water, MP, algae and daphnids). Each of the compartments mass is found in Table B, representing the experimental conditions for the depuration phase of the experiment. The mass of the *Daphnia* compartment corresponds to ten adult individuals, and the algal mass is the intended food concentration calculated using the cell number and their dry weight [4]. The masses of water, algae and microplastic were multiplied with two to account for the planned renewal of the media halfway during the depuration phase. Because the partitioning to algae and microplastic is expected to be fast [2,3], the system was assumed to have reached equilibrium before the water renewal, thus allowing a reasonable estimate of the PCB 209 distribution with double amount of the water, algae and plastic. Partition coefficients for PCB 209, between the different phases, were used to calculate the expected fractions in each compartment. As the polymer was unknown, we used different water-polymer partition coefficients to evaluate possible variability in the distribution due to the polymer composition.

Lipids were assumed as the sorbing phase in the daphnids and organic carbon as the sorbing phase in the algae. The organic carbon-water coefficient, according to Hansen et al. (1999) [5], was used to estimate partitioning to algae. The algae-water coefficient may differ from the distribution to organic carbon [2,6], but so can the coefficient between different algae species. As we were not able to find the distribution coefficient to PCB 209 for the algal species used in this experiment, a simplification by applying the organic carbon-water coefficient was deemed acceptable. The fraction of organic carbon was assumed to be 50% of the algae DW content.

Polymer-water coefficient for polydimethylsiloxane (PDMS) was according to Grant and colleagues [7] and the Lipid-PDMS coefficient for the same polymer from Jahnke et al. (2008) [8] (generic lipid value is used in the calculation). Using these references, we calculated the lipid-water, lipid-organic carbon and PDMS-organic carbon coefficients. Polymer-polymer coefficients from Gilbert et al (2016) [9], together with the PDMS-water coefficient by Grant et al. (2016) [7], were used to calculate polymer-water distribution for low-density polyethylene (LDPE) and silicone (O-ring, Polymicro and Altesil). Modelled PCB 209 polymer-water coefficients for LDPE and Altesil [10] were included for a comparison. The Polystyrene (PS)-water coefficient was according to Qian et al (2017) [11]. For polypropylene (PP), the polymer-water coefficient was estimated from LFER by plotting LogKow [12] against PP-water [13] and using the linear regression equation to determine PP-water coefficient for PCB 209. Polymer-lipid coefficients were calculated by using the lipid-water estimated from PDMS-Lipid [8] and PDMS-water [7] and polymer-water coefficients. Polymer-organic carbon coefficients were calculated similarly by using the water-organic carbon coefficient from Hanssen et al. (1999) [5] and polymer-water coefficients previously calculated for each polymer. The PCB 209 fractions in each compartment (f_a_) was calculated as (1) for control without plastic particles and as (2) for the samples with plastic particles.

$$f_{a}=\frac{C_{a}\times M_{a}}{C_{a}\times M_{a}+C_{b}\times M_{b}+C_{w}\times V_{w}} (1)$$

$$f_{a}=\frac{C_{a}\times M_{a}}{C_{a}\times M_{a}+C_{b}\times M_{b}+{C_{c}\times M_{c}+ C}_{w}\times V_{w}} (2)$$

Where C_a-c_ is the concentration of PCB 209 in each fraction (organic carbon, lipid and plastic particles) (mg kg^-1^) and M_a-c_ is the mass of the fraction in the system (kg), C_w_ is the concentration of PCB 209 in the water phase (mg L^-1^), and V_w_ is the volume of water (L). As the distribution of PCB 209 between a sorbing phase and water (D_a-w_; kg L^-1^) can be expressed as the ratio of the concentrations in a (C_a_; mg kg^-1^) and water (C_w_; mg L^-1^).

$$D_{a-w}=\frac{C_{a}}{C_{w}} (3)$$

The C_a-c_ in the equation can be replaced by D_a-c_ × C_w_ and rearranging gives (4) without plastic particles and (5) with plastic particles.

$$f_{a}=\frac{D_{a}\times M_{a}}{D_{a}\times M_{a}+D_{b}\times M_{b}+1\times V_{w}} (4)$$

$$f_{a}=\frac{D_{a}\times M_{a}}{D_{a}\times M_{a}+D_{b}\times M_{b}+D_{c}\times M_{c}+1\times V_{w}} (5)$$

The mass-balance estimates suggested that in the system without the microplastic, close to 70 % of PCB 209 remain in daphnids. Depending on the polymer type, exposure would either not affect (PS), have a minor effect (PP), or have a notable effect on the PCB 209 concentrations in the daphnids. When the experimental results were available, their comparison to the modelled values showed that our model overestimated the sorption capacity of the algae. In the test systems without microplastic – all PCB 209 was retained by the daphnids (119%). In the system with both algae and microplastic, the PCB 209 concentration in the daphnids was reduced to 45% compared to that at the beginning of the depuration phase. These dynamics suggests that these microplastic particles LogK_microplastic-water_ for PCB 209 was close to 7.

**Table B. Mass balance calculation.**

|  | **PCB 209** | *Daphnia*, Lipid Fraction of wet weight (mg) | Algae, Organic Carbon fraction of dry weight (mg) | MP (mg) | Water (kg) |
| --- | --- | --- | --- | --- | --- |
|  | **Mass** | 0.19 | 5.05 | 34.07 | 1 |
| **% distribution** | No MP | 67.19 | 32.47 |  | 0.35 |
|  | LDPE | 7.29 | 3.52 | 89.14 | 0.04 |
|  | LDPE^B^ | 0.80 | 0.39 | 98.81 | 0.00 |
|  | O-ring (silicon) | 17.25 | 8.34 | 74.33 | 0.09 |
|  | polymicro (silicon) | 11.23 | 5.43 | 83.28 | 0.06 |
|  | Altesil (silicon) | 11.75 | 5.68 | 82.52 | 0.06 |
|  | Altesil (silicon)^B^ | 12.05 | 5.83 | 82.06 | 0.06 |
|  | PDMS | 12.05 | 5.83 | 82.06 | 0.06 |
|  | PP | 48.13 | 23.26 | 28.36 | 0.25 |
|  | PS | 67.16 | 32.46 | 0.04 | 0.34 |

The distribution of PCB 209 (%) at the end of the depuration phase calculated for two experimental systems (1) without microplastic (water, algae and daphnids) and with microplastic (water, MP, algae and daphnids). Partition coefficients for PCB 209 between the different phases were used to calculate the expected fractions in each compartment. ^B^ Modelled polymer-water coefficients [10]

# References

1. Koelmans AA, Bakir A, Burton GA, Janssen CR. Microplastic as a Vector for Chemicals in the Aquatic Environment: Critical Review and Model-Supported Reinterpretation of Empirical Studies. Environ Sci Technol. 2016;50: 3315–3326.

2. Gerofke A, Kömp P, McLachlan MS. Bioconcentration of persistent organic pollutants in four species of marine phytoplankton. Environ Toxicol Chem. 2005;24: 2908–2917.

3. Wyman KD, O’Connors HB. Implications of short-term PCB uptake by small estuarine copepods (genus *Acartia*) from PCB-contaimined water, inorganic sediments and phytoplankton. Estuar Coast Mar Sci. 1980;11: 121–131.

4. Brown EJ, Harris RF. Kinetics of algal transient phosphate uptake and the cell quota concept 1. Limnol Oceanogr. 1978;23: 35–40.

5. Hansen BG, Paya-Perez AB, Rahman M, Larsen BR. QSARs for KOW and KOC of PCB congeners: A critical examination of data, assumptions and statistical approaches. Chemosphere. 1999;39: 2209–2228.

6. Sobek A, Gustafsson Ö, Hajdu S, Larsson U. Particle−Water Partitioning of PCBs in the Photic Zone:  A 25-Month Study in the Open Baltic Sea. Environ Sci Technol. 2004;38: 1375–1382. 7. Grant S, Schacht VJ, Escher BI, Hawker DW, Gaus C. Experimental Solubility Approach to Determine PDMS–Water Partition Constants and PDMS Activity Coefficients. Environ Sci Technol. 2016;50: 3047–3054.

8. Jahnke A, McLachlan MS, Mayer P. Equilibrium sampling: Partitioning of organochlorine compounds from lipids into polydimethylsiloxane. Chemosphere. 2008;73: 1575–1581.

9. Gilbert D, Witt G, Smedes F, Mayer P. Polymers as Reference Partitioning Phase: Polymer Calibration for an Analytically Operational Approach To Quantify Multimedia Phase Partitioning. Anal Chem. 2016;88: 5818–5826.

10. Smedes F, Geertsma RW, Zande T van der, Booij K. Polymer−Water Partition Coefficients of Hydrophobic Compounds for Passive Sampling: Application of Cosolvent Models for Validation. Environ Sci Technol. 2009;43: 7047–7054.

11. Qian J, Jennings B, M. Cwiertny D, Martinez A. Emerging investigator series: development and application of polymeric electrospun nanofiber mats as equilibrium-passive sampler media for organic compounds. Environ Sci Process Impacts. 2017;19: 1445–1456.

12. Hawker DW, Connell DW. Octanol-water partition coefficients of polychlorinated biphenyl congeners. Environ Sci Technol. 1988;22: 382–387.

13. Mato Y, Isobe T, Takada H, Kanehiro H, Ohtake C, Kaminuma T. Plastic Resin Pellets as a Transport Medium for Toxic Chemicals in the Marine Environment. Environ Sci Technol. 2001;35: 318–324.
