# Supplementary material for: Microplastic-mediated transport of PCBs? A depuration study with Daphnia magna
Source: PLoS One. 2019 Feb 19;14(2):e0205378. doi: 10.1371/journal.pone.0205378 (PMC6380591; doi:10.1371/journal.pone.0205378)
Supplement: S3 Text — (DOCX) [file pone.0205378.s003.docx]

# S3 Text. Quality Assurance for PCB analysis

The limit of quantification was 0.025 μg g^-1^ DW *Daphnia* and *Spirulina*, defined as a 5-fold peak over the chromatographic noise. In the PCB analyses of *Spirulina* powder and *Daphnia magna* samples, blanks were included in all runs and showed no peaks. ^13^C-PCB128 and ^13^C-PCB209 were used as internal standards. The recoveries of the internal standards were 65±19% for ^13^C-PCB128 and 102±32% for ^13^C-PCB209. A series (n=10) of spiked daphnids were also analysed with the following recovery values: PCB18: 81±9.1%, PCB40: 82±7.9%, PCB128: 72±14%, and PCB209: 59±12%. The extraction efficiency was estimated to be satisfactory.

**Table C. Monitored ions.**

| **Compound** | **Monitored ions (m/z)** | **Type** |
| --- | --- | --- |
| PCB 18 | 256^a^, 258 | Analyte |
| PCB 40 | 292^a^, 290 | Analyte |
| PCB 128 | 360^a^, 362 | Analyte |
| PCB 209 | 498^a^, 500 | Analyte |
| C_13_-PCB128 | 372 ^a^ | Isotope, Internal standard |
| C_13_-PCB209 | 510 ^a^ | Isotope, Internal standard |
| PCB 52 | 292 ^a^, 290 | Volumetric standard |

^a^Quantifying ion
