# Supplementary material for: Microplastic-mediated transport of PCBs? A depuration study with Daphnia magna
Source: PLoS One. 2019 Feb 19;14(2):e0205378. doi: 10.1371/journal.pone.0205378 (PMC6380591; doi:10.1371/journal.pone.0205378)
Supplement: S4 Text — (DOCX) [file pone.0205378.s004.docx]

# S4 Text. Quality Assurance for elemental composition analysis

Individual *D. magna* grown in the same conditions and fed the same type and quantity of food as the animals used in the experiment were used to determine precision of the elemental composition measurements. The analytical precision calculated as the standard deviation of a series of measurements (n=9) for the daphnids was 0.2 % for nitrogen (mean value 8.7 %) and 0.9 % for carbon (mean value 46.7 %).
