# Supplementary material for: Microplastic-mediated transport of PCBs? A depuration study with Daphnia magna
Source: PLoS One. 2019 Feb 19;14(2):e0205378. doi: 10.1371/journal.pone.0205378 (PMC6380591; doi:10.1371/journal.pone.0205378)
Supplement: S5 Text — (DOCX) [file pone.0205378.s005.docx]

# S5 Text. Polymer characterization by Fourier Transform Infrared (FTIR) Spectroscopy

FTIR spectra were recorded for two different types of MPs. Both were acquired from Cospheric (Goleta, USA) and contained the same fluorophore, however one was made of a polyethylene (PE), while no information regarding the other was provided by the supplier. The resulting spectra differ (Fig. S1). The dominant bands of the PE sample (red spectra) are due to the CH_2_ stretching and deformation vibrations and will not be discussed further. Many of the other bands in this spectrum are also found in the spectrum of the unknown fluorescent polymer (blue spectra) and can therefore be assigned to the fluorophore. They are much weaker in the PE spectrum, which seems to be caused by a lower fluorophore content in this sample. This is also suggested by its approximately 10-fold lower fluorescence intensity. Regarding the nature of the fluorophore, the absence of clear absorbance bands from aromatic and alkene compounds in the PE spectrum is telling. These characteristic bands are those from aromatic and heterocyclic CH stretching vibrations in the 3100-3000 cm^-1^ region (missing or very weak in the PE spectrum) and summation bands of the out-of-plane CH bending vibrations of aromatic compounds in the 2000 to 1650 cm^-1^ region (missing in both spectra). Most of these compounds have also an absorption band above 1590 cm^-1^ where only a weak shoulder is observed near 1620 cm^-1^. This seems to exclude an organic molecule with extended -electron system as a major component in our samples and points to quantum dots as the fluorophores. Quantum dots are often coated (capped) with carboxylate groups, the antisymmetric stretching vibration of which could account for the band near 1530 cm^-1^ in both samples. The absorption of this vibration depends on the coordination of the carboxylate group^3^ and positions close to those observed in our two samples have been observed for quantum dots: 1534 cm^-1^ ^4^ and 1528 cm^-1^.^5^ The following observations support the above assignment: (i) the band is strong and broad, which is indicative of a polar group that interacts with its environment. (ii) The band position is different for the PE sample and the unknown polymer sample, although the fluorophore is the same in both. This is in line with a location of the carboxylate groups on the surface of the quantum dots where they interact in different ways with the environment because they are embedded in different polymers. (iii) The band position of the unknown polymer sample shifts by 10 cm^-1^ to lower wavenumbers as the sample is set under pressure with the piston of the ATR unit (final pressure approx. 1.3 × 10^7^ Pa = 130 atm, Anders Nilsson, Bruker, personal communication). The band shifts partly back as the pressure is relieved. All other bands are not or much less affected. In contrast to the unknown polymer sample, the PE sample exhibits only a very minor shift. Again, this observation is in line with an assignment of the ~1530 cm^-1^ band to a vibration that is sensitive to changes in its environment.

The absorption of the symmetric stretching vibration is expected near 1400 cm^-1^. Here, the band at 1374 cm^-1^ of the unknown polymer sample exhibits a similar behaviour as the band at 1528 cm^-1^: it shifts down by 3 cm^-1^ upon application of pressure and partly relaxes back as the pressure is relieved. Therefore we tentatively assign this band to the symmetric stretching vibration of the carboxylate groups that also absorb near 1530 cm^-1^. The bands at 1590 and 1410 cm^-1^ might also be caused by carboxylates which are coordinated differently than the ones discussed so far and which are not as sensitive to pressure. All three bands (1590, 1410, and 1374 cm^-1^) have slightly different positions in the two samples.

It is not possible to identify the polymer component in the sample with the unknown polymer. Its spectrum seems to be completely dominated by the fluorophore component and no bands can unambiguously be assigned to the polymer component. This difficulty is also due to the much lower fluorophore content in our PE reference sample. We can conclude that the sample with unknown composition (polymer plus fluorophore) seems to contain NH or OH groups (absorption at 3412 cm^-1^), traces of aromatic, alkene, epoxy or cyclopropane groups (band profile around 3063 cm^-1^), and more methyl than methylene groups (CH stretching vibrations in the 3000-2800 cm^-1^ range). The characteristic bands of some common polymers were not detected, like those of PE, PE terephthalate, polyamide, polyester, polypropylene, polystyrene, and polyurethane. The polymer content is considerably less than in the PE sample.


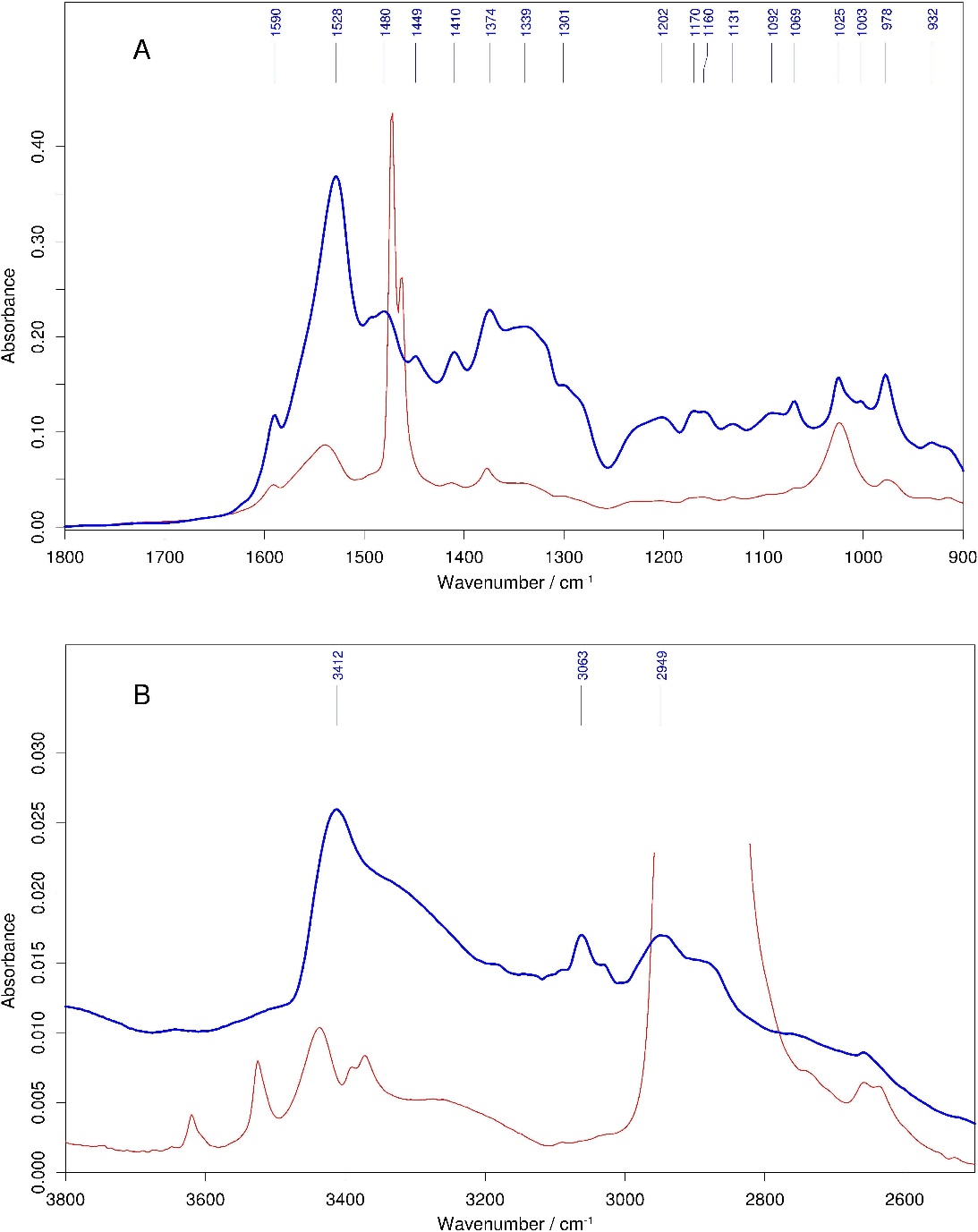


**Fig A**. **The absorbance spectra of the unknown polymer used in this study and of PE containing the same fluorophore**. The unknown polymer is depicted in blue and PE in red. (A) Spectral range 1800-900 cm^-1^, the PE spectrum was multiplied with a factor of 3. (B) Spectral range 3800- 2500 cm^-1^. The spectra were shifted and the region of the CH stretching vibrations of PE was cut out for a clearer presentation. In contrast to panel A, the PE spectrum was not enlarged in panel B.
